# Type I interferon and pattern recognition receptor signaling following particulate matter inhalation

**DOI:** 10.1186/1743-8977-9-25

**Published:** 2012-07-09

**Authors:** Aaron Erdely, James M Antonini, Rebecca Salmen-Muniz, Angie Liston, Tracy Hulderman, Petia P Simeonova, Michael L Kashon, Shengqiao Li, Ja K Gu, Samuel Stone, Bean T Chen, David G Frazer, Patti C Zeidler-Erdely

**Affiliations:** 1 Pathology and Physiology Research Branch, Health Effects Laboratory Division, National Institute for Occupational Safety and Health, Morgantown, WV, USA; 2 Laboratory for Occupational Cardiovascular Toxicology, Health Effects Laboratory Division, National Institute for Occupational Safety and Health, Morgantown, WV, USA; 3 Toxicology and Molecular Biology Branch, Health Effects Laboratory Division, National Institute for Occupational Safety and Health, Morgantown, WV, USA; 4 Biostatistics and Epidemiology Branch, Health Effects Laboratory Division, National Institute for Occupational Safety and Health, Morgantown, WV, USA; 5 NIOSH/HELD/PPRB, 1095 Willowdale Rd, MS-2015, Morgantown, WV, 26505-2888, USA

**Keywords:** Microarray, Welding, Interferon regulatory factor 7, Cardiovascular disease, Chromium, Biomarker, Pattern recognition receptor, Whole blood cell gene expression, Aorta, Inhalation

## Abstract

**Background:**

Welding, a process that generates an aerosol containing gases and metal-rich particulates, induces adverse physiological effects including inflammation, immunosuppression and cardiovascular dysfunction. This study utilized microarray technology and subsequent pathway analysis as an exploratory search for markers/mechanisms of *in vivo* systemic effects following inhalation. Mice were exposed by inhalation to gas metal arc – stainless steel (GMA-SS) welding fume at 40 mg/m^3^ for 3 hr/d for 10 d and sacrificed 4 hr, 14 d and 28 d post-exposure. Whole blood cells, aorta and lung were harvested for global gene expression analysis with subsequent Ingenuity Pathway Analysis and confirmatory qRT-PCR. Serum was collected for protein profiling.

**Results:**

The novel finding was a dominant type I interferon signaling network with the transcription factor *Irf7* as a central component maintained through 28 d. Remarkably, these effects showed consistency across all tissues indicating a systemic type I interferon response that was complemented by changes in serum proteins (decreased MMP-9, CRP and increased VCAM1, oncostatin M, IP-10). In addition, pulmonary expression of interferon α and β and *Irf7* specific pattern recognition receptors (PRR) and signaling molecules (*Ddx58*, *Ifih1*, *Dhx58*, ISGF3) were induced, an effect that showed specificity when compared to other inflammatory exposures. Also, a canonical pathway indicated a coordinated response of multiple PRR and associated signaling molecules (*Tlr7*, *Tlr2*, *Clec7a*, *Nlrp3*, *Myd88*) to inhalation of GMA-SS.

**Conclusion:**

This methodological approach has the potential to identify consistent, prominent and/or novel pathways and provides insight into mechanisms that contribute to pulmonary and systemic effects following toxicant exposure.

## Introduction

Welding represents an occupation that employs over 400,000 workers fulltime in the United States and over 5 million welders worldwide. Welding uses a consumable electrode wire to join two pieces of metal at high temperatures. The process generates an aerosol containing gases and a metal-rich particulate matter, known as the fume [1]. Therefore, an occupational health risk is present. The primary exposure route is by inhalation, and adverse effects of welding exposure in the lung have been shown in multiple human and animal studies [1-3]. In addition, deleterious effects are not confined to the lung. Epidemiological studies have shown an increased risk for ischemic heart disease in welders [4-6]. Subsequent human and animal studies have shown systemic inflammation and oxidative stress, vascular dysfunction, autonomic imbalance and advanced progression of atherosclerosis as a result of welding fume exposure [7-11]. Data also suggest that welders are immune compromised and more prone to bronchitis and pneumonia [1]. Indeed, animal studies show localized pulmonary immunosuppression following welding fume exposure [2,12,13].

Because pulmonary exposures are associated with extrapulmonary effects, such as cardiovascular dysfunction, systemic markers related to mechanisms of exposure have the potential to be monitored. Previous studies by our group showed a close crosstalk occurs between the pulmonary inflammatory response and the periphery [14]. Particularly, acute increases in blood inflammatory gene expression, altered leukocyte differentials and increased serum inflammatory proteins were a direct reflection of the ongoing pulmonary response following carbon nanotube (CNT) exposure [14,15]. These studies demonstrated methodologies which can be applied to assess potential systemic outcomes from a pulmonary exposure.

Occupationally derived particulate exposure levels may produce more subtle effects compared to higher dose or bolus lung instillation protocols, particularly when investigating systemic effects. Therefore, the generation of comprehensive transcriptome datasets by DNA microarray, along with gene network analysis, may prove valuable as a preliminary search for systemic mechanisms after welding fume exposure. In this study, microarray-derived gene networks from circulating whole blood cells, aorta and lung were compared to determine related biological signaling following inhalation of gas metal arc – stainless-steel (GMA-SS) welding fume in mice. Also, the applicability of this methodological approach for determining specific exposure-induced responses was examined.

## Results

### Welding fume characteristics and alveolar deposition

**Figure 1:**
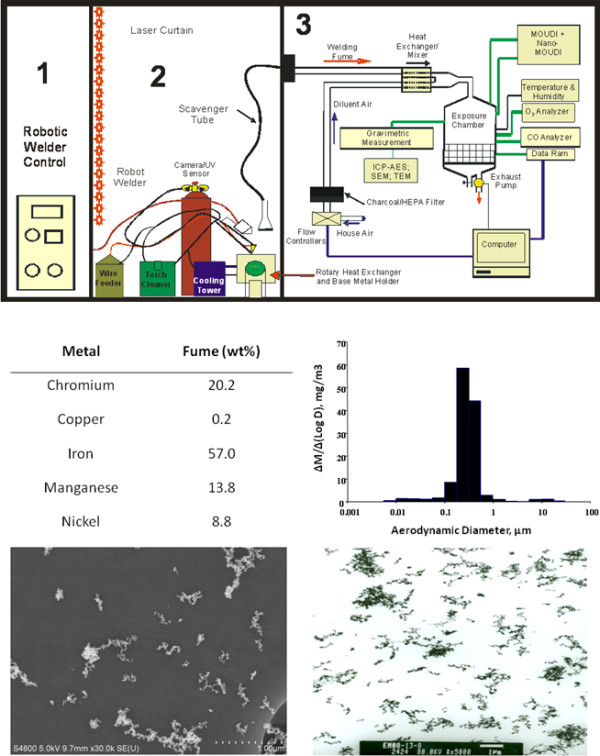
**Characterization of the welding fume exposure.** Diagram of the NIOSH welding inhalation system (upper panel): 1) Computer control and power source, 2) robotic welder and accompanying hardware necessary for fume generation and 3) exposure chamber and characterization devices. The middle left panel represents the metal content of the GMA-SS fume. The middle right panel shows the particle size distribution of the generated fume (M = mass concentration and D = aerodynamic diameter). The bottom panels show a scanning electron micrograph (left) and transmission electron micrograph (right) of the welding fume.

A diagrammatic description of the robotic welder used to generate fume for inhalation exposure and the components of the GMA-SS fume are shown in Figure 1. The fume consists primarily of iron, chromium, manganese, nickel and copper with trace amounts of silicon, aluminum and vanadium (Figure 1). The metal solubility in this type of fume is low (0.006 soluble to insoluble ratio). Representative micrographs of the GMA-SS fume are shown in Figure 1, and the mass median aerodynamic diameter was 0.255 μm [2]. Earlier studies have shown that a single exposure at our experimental conditions results in ~8.26 μg alveolar deposition in the lung. Comparing to human exposures this value represents 2–3.5 times the previous threshold limit value of 5 mg/m^3^ for total welding fume and 5.5-8.5 times the permissible exposure limit value of 5 μg/m^3^ for hexavalent chromium (Cr^VI^) [8]. Since welding may occur in poorly ventilated areas and measurements at the worker’s breathing zone can exceed recommended exposure limits [16,17], the exposure regimen in this study is relevant to an occupational exposure.

### Hierarchical clustering

Shown in Additional file 1: Figure S1 is the heatmap of differentially expressed genes in the blood from C57BL/6 J mice at 4 hr post-exposure to air or GMA-SS fume. Similar heatmaps were generated for aorta and lung (data not shown). Overall, expression patterns of the analyzed genes were more similar within exposure groups rather than between exposure groups. This indicates that a good consistency across samples was found on the individual arrays. In the blood, the annotated genes resulted in two distinct subclusters between the welding fume-exposed and air groups using our statistical criteria (p<0.05 and fold change +/− 1.1).

### Alterations in gene expression by microarray

#### Blood

**Figure 2:**
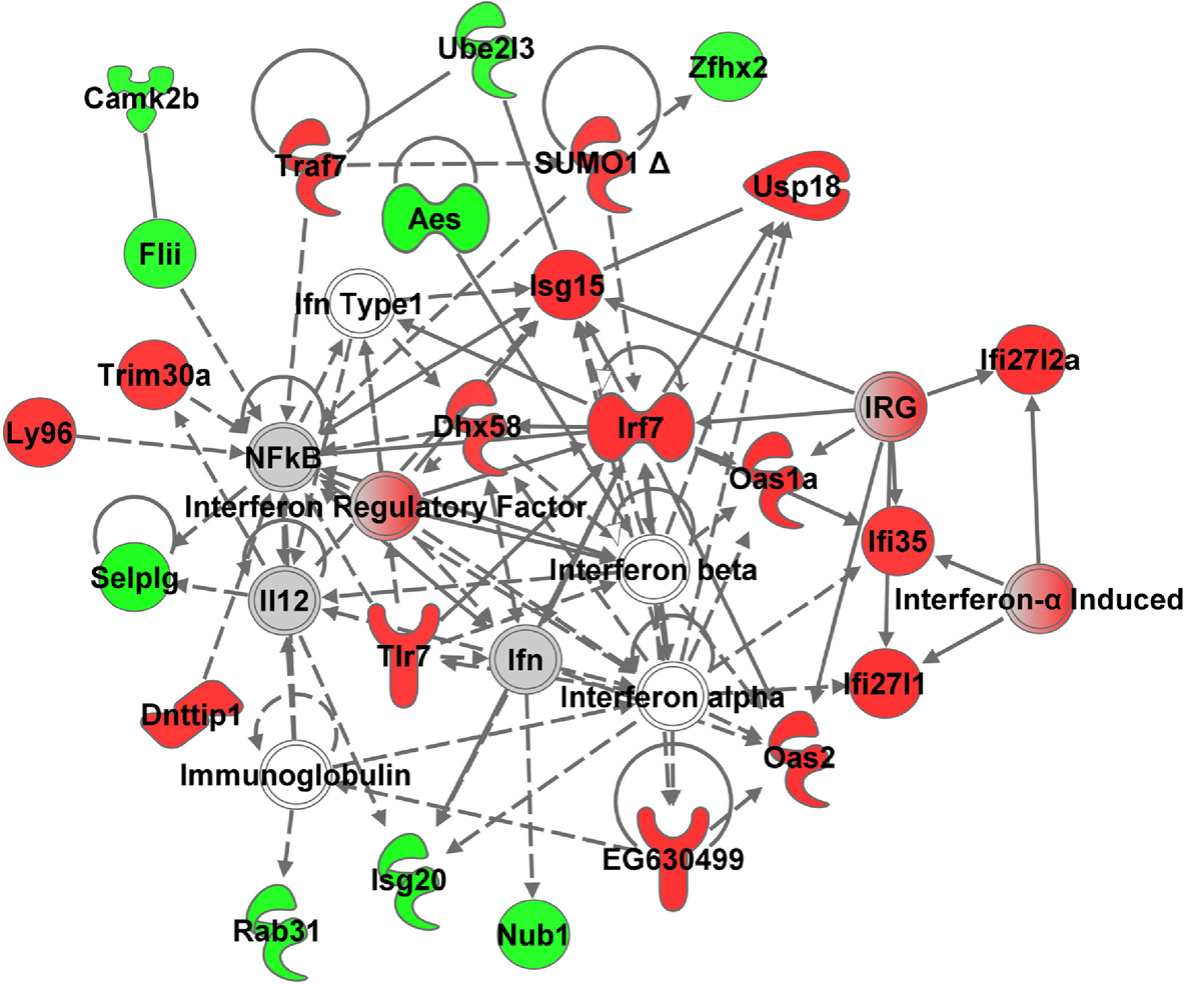
**Top molecular network in whole blood cells 28 d after GMA-SS inhalation exposure.** Gene network analysis by IPA indicated a significant type I interferon response following exposure. The network contained 25 of 35 focus molecules with a score of 44. A score of 44 means there is a 1 in 10^44^ chance of this network occurring by random chance. Intensity of the red (upregulated) or green (downregulated) color indicates level of gene expression. The white color indicates a predicted molecule incorporated from the Ingenuity knowledge base. Gray represents a molecule present in the dataset, but one that did not meet the specified cutoff criteria.

**Figure 3:**
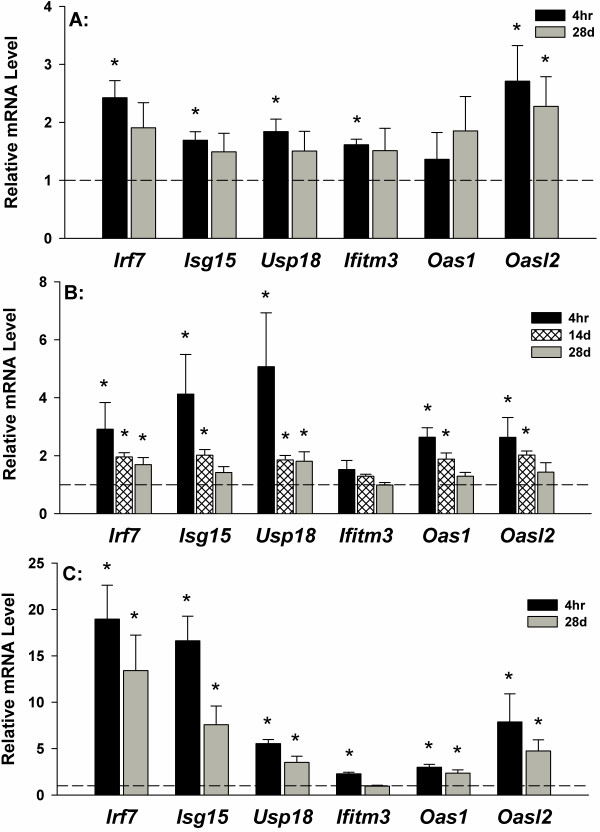
**Confirmation of microarray by qRT-PCR.** Relative mRNA expression levels of type I interferon related genes are shown in whole blood cells (**A**), aorta (**B**) and lung (**C**) in reference to sham (dotted line). *p<0.05 vs sham.

Microarray analysis was performed on whole blood samples, aorta and lung at 4 hr and 28 d following the 10 d of inhalation exposure. The initial aim was to evaluate whole blood cells for a gene network signature of GMA-SS exposure. Utilizing the specified analysis criteria, 90 and 211 genes eligible for network analysis by Ingenuity Pathway Analysis (IPA) were altered due to inhalation of GMA-SS fume at 4 hr and 28 d, respectively. The networks, “organismal development, infection mechanism, antimicrobial response” and “antimicrobial response, inflammatory response, post-translational modification” were the most significant networks at 4 hr (Additional file 1: Figure S2) and 28 d (Figure 2), respectively. Both networks consisted of genes related to type I interferon signaling and a central component to the pathway was the transcription factor interferon regulatory factor 7 (*Irf7)* (Additional file 1: Figure S2 and Figure 2; Additional file 1: Table S1). This effect was evident in the top differentially regulated genes at 4 hr and 28 d post-exposure (Additional file 1: Table S2). *Irf7* and several type I interferon related genes including *Isg15*, *Usp18*, *Ifitm3*, *Oas1* and *Oasl2* were confirmed by qRT-PCR at these time points (Figure 3A). These effects were also most prominent in the functional analysis at 4 hr (data not shown) and 28 d (Additional file 1: Figure S3) post-exposure. The determination of *Irf7* as the central component was entirely subjective. This claim was supported by our interpretation of the IPA analysis (e.g. *Irf7* gene expression was the highest (4 hr) and second highest (28 d) expressed gene in the blood and was primary to the most significant IPA networks), consistency and level of expression with time and tissue (e.g. confirmatory quantitative real-time RT-PCR), and supportive information in the literature indicating IRF7 the master regulator of the type I interferon response [18]. Together these findings contribute to our claim of *Irf7* as a central signaling component induced by GMA-SS inhalation.

#### Aorta

**Figure 4:**
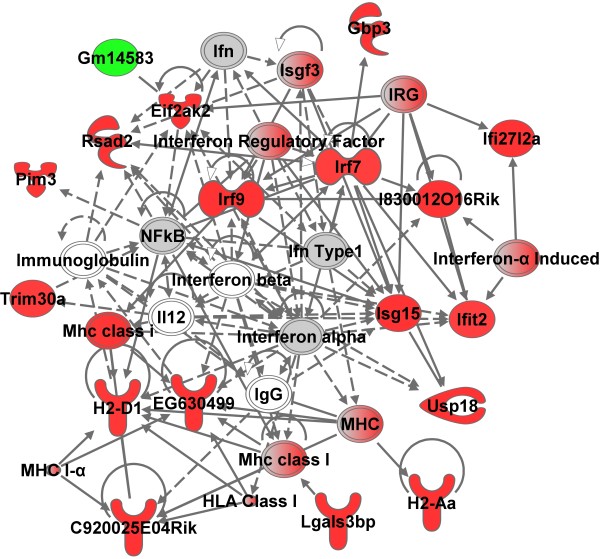
**A top molecular network in aorta at 4 hr after GMA-SS inhalation exposure.** Gene network analysis by IPA indicated a significant type I interferon response following exposure. The network contained 18 of 35 focus molecules with a score of 35. A score of 35 means there is a 1 in 10^35^ chance of this network occurring by random chance. Intensity of the red (upregulated) or green (downregulated) color indicates level of gene expression. The white color indicates a predicted molecule incorporated from the Ingenuity knowledge base. Gray represents a molecule present in the dataset, but one that did not meet the specified cutoff criteria.

Utilizing the analysis criteria, 90 and 165 genes eligible for network analysis by IPA were altered due to GMA-SS fume exposure at 4 hr and 28 d, respectively. At 4 hr, the top 2 networks were “antimicrobial response, inflammatory response, infection mechanism” and “antimicrobial response, inflammatory response, post-translational modification” showing similarity to the response in the whole blood cells (data not shown and Figure 4 respectively; Additional file 1: Table S3). The transcription factor *Irf7* was a central molecule in the response of the aorta. Confirmation by qRT-PCR of type I interferon related genes showed the effect was still prominent at 14 d post-exposure and resolving toward baseline by 28 d (Figure 3B). Similar to the blood, the functional analysis was a reflection of the network analysis described above (Additional file 1: Figure S4). At 28 d, 164 genes were eligible for network analysis; however, the above mentioned gene networks were not a significant part of the results (data not shown).

#### Lung

**Figure 5:**
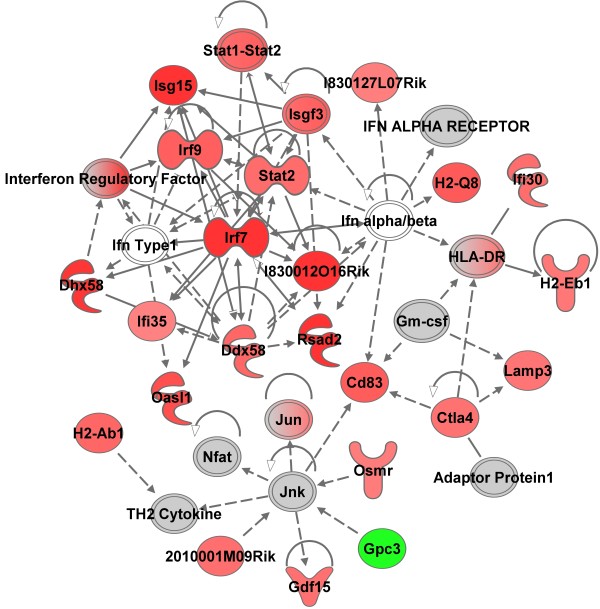
**A top molecular network in lung at 28 d after GMA-SS inhalation exposure.** Gene network analysis by IPA indicated a significant type I interferon response following exposure. The network contained 22 of 35 focus molecules with a score of 30. A score of 30 means there is a 1 in 10^30^ chance of this network occurring by random chance. Intensity of the red (upregulated) or green (downregulated) color indicates level of gene expression. The white color indicates a predicted molecule incorporated from the Ingenuity knowledge base. Gray represents a molecule present in the dataset, but one that did not meet the specified cutoff criteria.

A major hypothesis of our laboratory is that the systemic gene signature reflects the ongoing pulmonary response induced by a particle. Due to the known pulmonary toxicity of this exposure regimen [3], significant changes were expected in the lung tissue. Indeed, 439 and 368 genes eligible for network analysis by IPA were altered at 4 hr and 28 d post-exposure, respectively. A complete analysis of the global lung response was not the focus of this study, instead whether a type I interferon response was evident following analysis of the blood and aorta. In the lung at 4 hr and 28 d, four significant networks (two at 4 hr and 28 d) indicating infectious disease, antimicrobial response and infection mechanism were evident (Figure 5; Additional file 1: Figure S5 highlight two networks; Additional file 1: Table S4). Corresponding to the blood and aorta, a central component of the IPA networks was *Irf7*. Several of the genes were confirmed by qRT-PCR (Figure 3C).

### Serum protein profiling

A panel of 59 analytes was measured in the serum before the outcome of the microarray data was learned. The panel was composed mainly of inflammatory markers, and interferon alpha and beta were not included; however, several proteins from the panel complemented a systemic type 1 interferon response. C-reactive protein (CRP) and matrix metalloproteinase 9 (MMP-9) were decreased and oncostatin M (OSM) was increased at 4 hr (Additional file 1: Table S5). By 28 d post-exposure the changes included decreased immunoglobulin A (IgA) and a highly significant increase in vascular cell adhesion molecule-1 (VCAM-1). At both time points there was a general trend for increased serum inducible protein 10 (IP-10) (41% at 4 hr and 50% at 28d; Additional file 1: Table S5).

### Type I interferon and related pattern recognition receptor (PRR) induction

**Figure 6:**
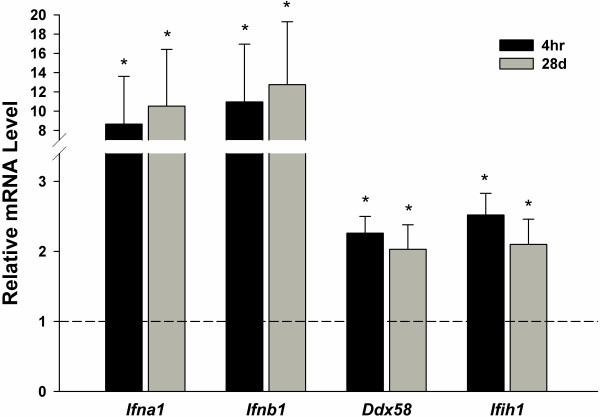
**Effects of GMA-SS inhalation exposure on pulmonary type I interferons and associated pattern recognition receptors.** Relative mRNA levels of interferon alpha (*Ifna* – recognizes interferon alpha 1, 5 and 6), interferon beta (*Ifnb* – recognizes interferon beta 1), and the pattern recognition receptors *Ddx58* (DEAD box polypeptide 58; RIG-I) and *Ifih1* (interferon induced with helicase C domain; MDA5) were increased in the lung in reference to sham (dotted line). *p<0.05 vs sham.

To complement *Irf7* induction, the expression of the type I interferon genes *Ifna1* (identifies interferon alpha 1, 5 and 6) and *Ifnb1* (interferon beta 1) and the intracellular PRR *Ddx58* (DEAD box polypeptide 58; RIG-I) and *Ifih1* (interferon induced with helicase C domain; MDA5) were evaluated by qRT-PCR. These genes were increased in the lung at 4 hr and 28 d post exposure (Figure 6). Another related intracellular PRR, *Dhx58* (DEXH box polypeptide 58; LGP2), was increased in the array data (2.355 fold induction p = 2.89E-09 at 4 hr and 2.157 fold induction p = 2.35E-08 at 28 d). Also, *Tlr7* was increased at both time points (1.271 fold induction p = 3.12E-04 at 4 hr and 1.369 fold induction p = 1.05E-05 at 28 d). IPA analysis also revealed a significant IRF canonical pathway “activation of IRF by cytosolic PRR” at 4 hr (data not shown) and 28 d (Additional file 1: Figure S6) illustrating both upstream and downstream signaling related to *Irf7* induction.

### Additional PRR induction

In addition to the specific IRF-associated PRR canonical pathway, IPA analysis showed a network of general PRR induction in the lung following welding fume inhalation exposure at 4 hr (data not shown) that was still evident 28 d (Additional file 1: Figure S10) post-exposure. Gene expression of several other PRR and associated signaling molecules was induced (Additional file 1: Figure S7; Additional file 1: Table S6). Microarray analysis indicated induction of *Il1b* and *Casp1* suggesting NALP3 activation. Confirmation by qRT-PCR of *Nlrp3* and associated genes are shown in Additional file 1: Figure S8; significant effects were seen at 28 d after exposure. In this study, IL-1β was not increased in the serum, but was increased in a previous exposure at 14 d post [8] suggesting a peak in the response may have occurred sometime between 4 hr and 28 d.

### Specificity of Irf7 signaling

**Figure 7:**
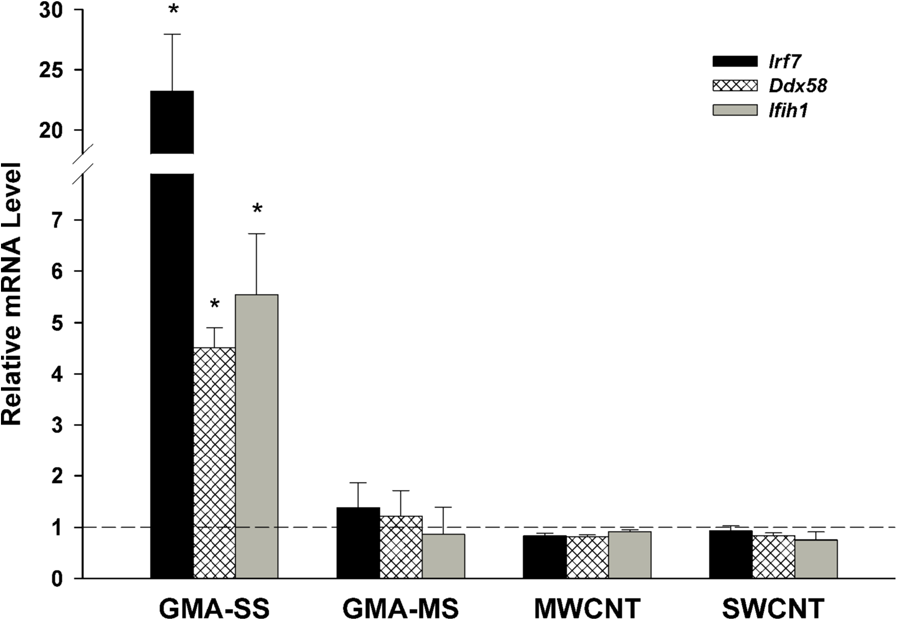
**Effects of multiple different pulmonary exposures on lung mRNA levels of *Irf7 *and associated pattern recognition receptors.** Lung RNA extracted from previously published exposures was assessed post-hoc with respect to the new findings from this study. Male C57BL/6 J mice were exposed to 340 μg of gas metal arc – stainless steel (GMA-SS), 340 μg gas metal arc – mild steel (GMA-MS), 40 μg of multi-walled carbon nanotubes (MWCNT), or 40 μg single-walled carbon nanotubes (SWCNT) (n = 5 or 6 per group for all exposures) by pharyngeal aspiration and lungs were harvested 24 hr post-exposure [15,19]. Relative mRNA levels of *Irf7* (interferon regulatory factor 7) and the pattern recognition receptors *Ddx58* (DEAD box polypeptide 58; RIG-I) and *Ifih1* (interferon induced with helicase C domain; MDA5) were increased in the lungs of GMA-SS exposed mice only in reference to sham (dotted line). *p<0.05 vs sham.

One question which arose from this methodological approach was the uniqueness of a consistent molecular signature, in this case *Irf7* and type I interferon signaling following GMA-SS inhalation exposure, in pulmonary and extrapulmonary compartments. To gain insight, previous experimental designs were examined for *Irf7*, *Ddx58* and *Ifih1* induction. A recent study by our group compared the pulmonary and systemic effects of SS and mild steel (MS) welding fume by pharyngeal aspiration in male C57BL/6J mice [19]. At 24 hr post-aspiration of 340 μg of GMA welding fume, analysis of the lungs showed the GMA-SS, but not GMA-MS, fume increased *Irf7*, *Ddx58* and *Ifih1* gene expression (Figure 7). Also, previous pulmonary exposures to 40 μg of multi-walled CNT or single-walled CNT in male C57BL/6J mice [15] showed no induction of *Irf7* or associated PRR (Figure 7). The exposures to both welding fume and CNT induced marked cytotoxicity, measured by bronchoalveolar lavage lactate dehydrogenase activity (Additional file 1: Figure S9A), a marked inflammatory response (Additional file 1: Figure S9B), and inflammatory cell influx [15,19] indicating a generalized response to inflammation was not likely contributing to the increase of these specific genes.

### Discussion and conclusion

The novel findings of this study include blood cell activation with dominant type I interferon signaling following 10 d of welding fume inhalation. Similar signaling pathways were found in the lung and, interestingly, in the aorta. Systemically, the response was still measurable 28 d after the exposure ceased. In addition, gene expression levels of several pattern recognition receptors and associated signaling molecules were increased, including those directly known to induce *Irf7*. These results showed an identifiable uniform network of expression in the blood, vasculature and lung with a level of specificity to SS welding fume when compared to other exposures.

Previously, pulmonary toxicity of this exposure regimen in a C57BL/6J mouse model was published and Additional file 1: Table S7 was adapted to illustrate the response to GMA-SS inhalation [3]. The fume exposure resulted in cytotoxicity and increased permeability, as shown by bronchoalveolar lavage (BAL) lactate dehydrogenase (LDH) activity and albumin, respectively (Additional file 1: Table S7). These effects were found 1 d after 10 d of inhalation exposure and there was no resolution at 28 d post-exposure. Polymorphonuclear leukocytes (PMN) were increased 1 d after exposure and showed further increased levels at 14 and 28 d (Additional file 1: Table S7). The exposure also increased total BAL lymphocytes and macrophages and this response was maintained throughout the time course (Additional file 1: Table S7). These data indicate a sustained inflammatory response with a delayed, but marked PMN influx. These changes were inconsistent with type I interferon signaling with time indicating that pulmonary injury and inflammatory cell influx was likely not a major contributor to the type I interferon signaling.

In this study, a single gene network was prominent and consistent between the lung, whole blood cells and aorta. This network was reflective of type I interferon signaling with the transcription factor *Irf7* as a central hub. Recent studies have indicated IRF7 as the master regulator of the type I interferon response meaning induction of interferon α and β are dependent on IRF7 [18]. This was supported by induction of interferon-α and -β gene expression in lungs from GMA-SS-exposed mice. Interestingly, the data also showed a similar response in the periphery as increased interferon signaling in both circulating blood cells and the aorta was measured. In support, decreased serum CRP and MMP-9, increased VCAM-1 and the general increase in IP-10 are all consistent with type I interferon therapy [20-24]. Also, OSM, an interleukin-6 family member that is released with and enhances the effects of type I interferons [25], was increased in the serum. Therefore, the primary response, which originates in the lung, provided an identifiable systemic gene network signature.

Type I interferons are a class of proteins involved in the innate immune response which can be induced by PRR. In this study, three cytoplasmic PRR in the RIG-I-like receptor family (*Ddx58*/RIG-I, *Ifih1*/MDA-5 and *Dhx58*/LGP2) were increased as a result of GMA-SS exposure. RIG-I and MDA-5 are known to induce IRF7 while LGP2 inhibits the other two receptors [26]. In addition, expression of *Tlr7*, a PRR with the potential to induce *Irf7*[26], was increased. Generally, PRR can be activated by pathogen-associated molecular patterns (PAMPs), danger-associated molecular patterns (DAMPs) and particles [27]. Of the three classes of activators, PAMPs seem the least likely as activation of the above described PRR can occur from single stranded RNA, intracellular viral RNA and synthetic dsRNA [27] that, even if present, would not retain integrity at the >4000 °C at which the welding fume is generated. Also, the results from this study do not favor DAMPs, at least acutely, given that other known pulmonary inflammatory agents did not increase *Irf7* gene expression or related PRR (Figure 7).

Recently, PRR have been shown to play a role following a particulate exposure and the study of associated mechanisms of activation and subsequent pulmonary and systemic effects is only beginning. Pulmonary exposures to silica or asbestos have been shown to activate the cytosolic PRR NALP3 inflammasome, a process resulting from particle uptake [28-30]. Knockout of the NALP3 inflammasome has been shown to reduce inflammation and fibrosis [28-30]. Pulmonary inflammation resulting from inhalation of PM for 20 weeks, instillation of residual oil fly ash or inhalation of cigarette smoke was attenuated with deficiency of *Tlr4*[31-33]. In this study, PRR including the RIG-I family members, *Tlr7*, *Tlr2*, *Clec7a*, *Nlrp3* related genes, and the scavenger receptor *Marco*, as well as associated signaling molecules, were increased. These results suggest a coordination of activated signaling PRR pathways which mediate a response to an inhaled, inflammatory particulate. Different extracellular and intracellular bacterial and viral pathogens are recognized by multiple PRR [27] and the same redundancy may be true for the pulmonary response to particulate exposures.

Particularly intriguing was that exposure to MS welding fume or CNT, known inflammatory exposures [14,15,19,34], did not increase *Irf7* or specifically related PRR. On the other hand, high dose (2.5 mg/mouse) instillation exposure to silica was recently shown to induce pulmonary *Irf7*[35]. The appearance that induction of *Irf7* was a direct effect of GMA-SS exposure, while certainly true acutely, was only addressed at 24 hr after a single exposure by aspiration. Therefore, induction of type I interferon signaling by other toxicants in a secondary fashion to repeated exposures and/or additional time points post-exposure cannot be excluded. While this is plausible, an alternate explanation suggests that silica exposure may result in direct activation of *Irf7* as C57BL/6J mice had increased *Irf7* gene expression at 3d, the earliest post-exposure time point examined [35].

In regards to specificity, a recent study by Nemec et al. showed that Cr^VI^ stimulates *Irf7* gene expression by stimulating Fyn phosphorylation of STAT1 and subsequent activation of interferon stimulated gene factor 3 (ISGF3) in human airway epithelial cells [36]. These effects were independent of type I interferon receptor activation. Of note, the induction of *Irf7* by silica was type I receptor dependent suggesting a different mechanism when compared to Cr^VI^. Since 0.26% by weight of GMA-SS is Cr^VI^[37], the potential mechanism of inhalation exposure includes direct activation of *Irf7* gene expression followed by induction of type I interferons initiating a positive feedback loop. In addition, ISGF3, a heterotrimeric complex consisting of STAT1, STAT2 and IRF9, was induced as illustrated in the IRF canonical pathway (Additional file 1: Figure S8). The signaling can also account for associated PRR induction because activation of IRF7 dependent type I interferon signaling increases RIG-I and MDA5 expression [26,38,39], although a direct mechanism cannot be excluded. Therefore, while some PRR may be activated in a nonspecific manner, such as general particle uptake, inflammatory and/or oxidative stress mechanisms (e.g. NALP3, toll-like receptors), some may be uniquely activated.

Does the increased type I interferon signaling contribute to the toxicological effects of GMA-SS? One potential role for *Irf7* is inflammation. For example, knockout of *Irf7* in a silica-exposed mouse model decreased chronic pulmonary inflammation [35], an effect known to occur after GMA-SS inhalation in an animal model [2,3]. Since pulmonary inflammation is linked to systemic inflammation and resultant adverse effects, such as cardiovascular dysfunction [40], *Irf7* may not only contribute to lung inflammation but to these other effects as well. In support, a recent study by Kampfrath et al. showed knockout of the PRR *Tlr4* not only reduced pulmonary inflammation, but also blunted systemic inflammation and vascular dysfunction following particulate matter inhalation [33]. Previous studies have shown that mice fed a high fat diet had increased *Irf7* and related type I interferon signaling in the aorta [41]. Also, an IRF7-dependent inflammatory network was associated with the pathogenesis of type I diabetes [42] and infection induced *Irf7* in endothelial cells may contribute to the development of vascular lesions [43]. These studies suggest that cardiovascular dysfunction and differential regulation of IRF7 may be linked. Currently, the role of IRF7 in the vasculature is unknown and additional studies are warranted.

Interestingly, the pulmonary and systemic induction of interferon seems counterintuitive to the reported immunosuppression following metal-rich fume exposure. In fact, this study showed decreased serum IgA 28 d after exposure ended, which supports a previous finding in human welders [44]. Human welders are more prone to develop bronchitis and pneumonia, and animal studies have shown the inability to clear *L. monocytogenes* after welding inhalation exposure [1,2,13]. Since GMA-SS, GMA-MS or CNT exposure induce localized immunosuppression [2,13,45], but only GMA-SS induced type I interferon signaling, a general compensatory upregulation in response to a dampened immune system seems unlikely. In fact, the exact opposite may be true. Type I interferon receptor knockout mice are resistant to *L. monocytogenes* exposure [46]. This provides a direct mechanistic link of Cr^VI^ exposure to a suppressed lung defense via IRF7 and also can explain why *L. monocytogenes*, under exactly the same exposure conditions, produced a greater effect in SS as compared to MS fumes [2,13]. Finally, chronic elevation of type I interferons, although utilized therapeutically, has the potential to cause immunosuppression [47,48], supporting the effects found in humans and animal studies.

The aim of this study was to analyze lung, whole blood cells and the vasculature for a related, identifiable molecular pathway related to GMA-SS exposure. Our expectations were exceeded by the consistency of the response that also proved to have some specificity. Our data, combined with Nemec et al. (2010), suggests the IRF7-dependent signaling network was conserved across species and consistent over time and between tissues. Therefore, utilization of this methodological approach has the potential to identify consistent, prominent and/or novel pathways induced by a toxicant exposure. The gene network(s) may then be manipulated to address direct pulmonary and extrapulmonary effects. Even if the identified network(s) does not contribute to marked exposure effects, it could be used for exposure monitoring. In this study, the systemic measure of *Irf7* and type I signaling become a surrogate marker of GMA-SS fume exposure.

## Methods

### Study design and exposure

Male C57BL/6J mice from Jackson Laboratory (Bar Harbor, ME) were used in this study. All mice were provided food and tap water *ad libitum* in ventilated cages in a controlled humidity and temperature environment with a 12 hr light/dark cycle. Animal care and use procedures were conducted in accordance with the “PHS Policy on Humane Care and Use of Laboratory Animals” and the “Guide for the Care and Use of Laboratory Animals” (NIH publication 86–23, 1996). These procedures were approved by the National Institute for Occupational Safety and Health Institutional Animal Care and Use Committee.

C57BL/6J mice, 6 weeks of age, were exposed by inhalation to gas metal arc-stainless steel (GMA-SS) welding fume at 40 mg/m^3^ for 3 hr/d for 5 d a week for two weeks (exposures day 1–5 and 8–12). The welding fume generation system and characterization for this particular fume has been extensively described [2,49]. Mice were sacrificed by carbon dioxide asphyxiation at 4 hr (n = 4 air; n = 4 GMA-SS), 14 d (n = 4 air; n = 4 GMA-SS) and 28 d (n = 8 air; n = 8 GMA-SS) post-exposure. Whole blood cells, aorta and lung were collected for global expression analysis at 4 hr and 28 d post exposure. The aorta was collected from all time points. All samples were stored at −80 °C prior to analysis. Serum was collected 4 hr and 28 d post exposure and sent to Myriad/RBM (Austin, TX) for protein profiling by multiplex immunoassay RodentMAP v2.0.

### Gene expression

RNA was isolated from whole lung homogenates using the TRIzol (Invitrogen, Carlsbad, CA) method and then cleaned according to the manufacturer’s instructions using a RNeasy Mini Kit (Qiagen, Valencia, CA, USA). The aorta was homogenized with a Qiagen Tissue Lyser and RNA was isolated using the RNeasy fibrous tissue mini kit (Qiagen). Whole blood RNA was isolated using the Mouse RiboPure^TM^ Blood RNA Isolation Kit (Ambion, Austin, TX, USA) according to manufacturer’s directions and globin RNA was removed using the GLOBINclear^TM^ kit (Ambion). A 2 μl aliquot of each RNA sample was quantified using a NanoDrop ND-1000 spectrophotometer (NanoDrop Technologies, Inc., Wilmington, DE, USA) and quality was assessed on the Agilent 2100 Bioanalyzer (Agilent Technologies, Palo Alto, CA, USA).

Labeled cRNA, from an input RNA of 375 ng, was prepared according to the manufacture’s protocol, using the Illumina TotalPrep RNA Amplification Kit (Ambion, Catalog #IL1791) for hybridization to the arrays. The labeled cRNA samples were then assessed for quality and quantity. To ensure consistency for the array hybridization, all cRNA samples for each time point were quantified at the same time. The MouseRef-8 v2.0 beadchip contains >24,000 well annotated RefSeq transcripts and allows eight samples to be interrogated in parallel. After a 20 hr hybridization period at 58 °C, the beadchips were scanned using an Illumina BeadStation 500 G - BeadArray Reader (Illumina, Inc., San Diego, CA, USA). The microarray data were deposited to Gene Expression Omnibus (GEO) (http://www.ncbi.nlm.nih.gov/geo/) and are accessible through accession number GSE34056.

Confirmation of microarray results by quantitative real-time reverse transcription polymerase chain reaction (qRT-PCR) was done as follows. Evaluation of gene expression was determined by standard 96-well technology using the StepOne™ (Applied Biosystems, Carlsbad, CA, USA) with pre-designed Assays-on-Demand™ TaqMan® probes and primers (Applied Biosystems). Using 96 well plates, one μg of total RNA was reverse transcribed using random hexamers (Applied Biosystems) and Superscript III (Invitrogen, Carlsbad, CA). Nine μl of cDNA (1/10) was then used for gene expression determination. Hypoxanthine-guanine phosphoribosyltransferase was used as an internal reference. Relative gene expression was calculated using the comparative threshold method (2^-ΔΔCt^) with vehicle treated mice serving as the reference group [50].

### Hierarchial clustering and heatmap generation

Heatmaps were generated using the ‘pheatmap’ package in R using the correlation distance measure and complete linkage for the construction of the dendrograms. Genes with a nominal p-value of less than 0.05 and a fold change of at +/− 1.1 were included in the map.

### Statistics and data analysis strategy

Statistical analysis procedures for whole microarray datasets have been extensively described [51]. Briefly, samples were imported into illumina® Beadstudio 3.0.19.0 and reference, hybridization control, stringency and negative control genes were checked for proper chip detection. Beadarray expression data were then exported with mean fluorescent intensity across like beads and bead variance estimates into flat files for subsequent analysis. Illumina BeadArray expression data were analyzed in Bioconductor using the ‘lumi’ and ‘limma’ packages. Gene lists containing group means of expression, p-values and standard fold changes were utilized as input for subsequent bioinformatics analysis.

The networks and functional analyses were generated through the use of Ingenuity Pathways Analysis (Ingenuity® Systems, http://www.ingenuity.com). Whole datasets containing gene identifiers and corresponding expression values were uploaded into the application and a core analysis was done. Each identifier was mapped to its corresponding object in Ingenuity's Knowledge Base. In this study the following analysis criteria were utilized: whole blood cells (fold change = 1.1, p<0.05), aorta (fold change = 1.1, p<0.05) and lung (1.3 fold change, p<0.05). The criteria were chosen because it provided the best representation of the IPA analysis. For the whole blood cells and aorta, additional analysis including a more stringent p value (p<0.02), higher fold change (1.3 fold) or all significant genes (p<0.05) showed a significant type I interferon response (data not shown). Previous analysis showed that the chosen criteria for the lung were optimal [51]. These differentially regulated genes, called Network Eligible Molecules, were overlaid onto a global molecular network developed from information contained in Ingenuity’s Knowledge Base. Networks of these molecules were then algorithmically generated based on their connectivity.

The data presented in this manuscript was selected to represent the type I interferon/IRF7 response induced by GMA-SS inhalation since the signaling was prominent in the lung and systemically. An additional manuscript is being written to include a broader analysis of the microarray results and will include time comparisons, a broader interpretation of the signaling networks generated for the lung, blood and aorta, and a detailed analysis of the pulmonary response.

The Functional Analysis identified the biological functions and/or diseases that were most significant to the data set. Molecules from the dataset that met the cutoff criteria defined above and were associated with biological functions and/or diseases in Ingenuity’s Knowledge Base were considered for the analysis. Right-tailed Fisher’s exact test was used to calculate a p-value determining the probability that each biological function and/or disease assigned to that data set is due to chance alone. Canonical pathways analysis identified the pathways from the Ingenuity Pathways Analysis library of canonical pathways that were most significant to the data set. Molecules from the data set that met the cutoff criteria defined above and were associated with a canonical pathway in Ingenuity’s Knowledge Base were considered for the analysis. The significance of the association between the data set and the canonical pathway was measured in 2 ways: 1) A ratio of the number of molecules from the data set that map to the pathway divided by the total number of molecules that map to the canonical pathway is displayed. 2) Fisher’s exact test was used to calculate a p-value determining the probability that the association between the genes in the dataset and the canonical pathway is explained by chance alone.

Since the consistent signaling pathway was evident in multiple tissues at more than one time point with varying stringency, confirmed by qRT-PCR and induced by a completely separate exposure regimen (Figure 7), the representation and interpretation of the results are thoroughly supported. For analysis other than microarray, all data are presented as means ± standard error. Since each time point contained respective controls and were harvested separately, all data were compared as sham versus exposed by Student’s *t*-test at each time point. Differences were considered statistically significant at p<0.05.

## Competing interests

The authors declare that they have no competing interests

## Authors’ contributions

AE analyzed the data output and drafted the manuscript. MLK, SL, and JKG conducted the statistical analysis. SS, BTC, DGF conducted the inhalation exposures. RSM, AL and TH performed confirmatory molecular analysis of the microarray results. PCZE processed the microarray samples and performed the Ingenuity pathway analysis. AE, JMA, PPS and PCZE conceived and designed the study. All authors have read and approved of the final version of the manuscript.

“The findings and conclusions in this report are those of the author(s) and do not necessarily represent the views of the National Institute for Occupational Safety and Health”

## Supplementary Material

Additional file 1 Contains additional data to supplement the microarray and pathway analysis data presented in the results section. Click here for file
