# Supplementary material for: Type I interferon and pattern recognition receptor signaling following particulate matter inhalation
Source: Part Fibre Toxicol. 2012 Jul 9;9:25. doi: 10.1186/1743-8977-9-25 (PMC3537608; doi:10.1186/1743-8977-9-25)

Additional file 1

Type I interferon and pattern recognition receptor signaling following particulate matter inhalation

Aaron Erdely (efi4@cdc.gov)*'†, James M. Antonini (jga6@cdc.gov)*, Rebecca Salmen-Muniz (zvj4@cdc.gov)*'†, Angie Liston (listonangie@yahoo.com)[‡], Tracy Hulderman (tmh7@cdc.gov)*'†, Petia P. Simeonova (pns6@cdc.gov)[‡,¶], Michael L. Kashon (mqk1@cdc.gov)[§], Shengqiao Li (lis2@upmc.edu)[§], Ja K. Gu (gum4@cdc.gov)[§], Samuel Stone (zhf9@cdc.gov)*, Bean T. Chen (bdc4@cdc.gov)*, David G. Frazer (dgf1@cdc.gov)* and Patti C. Zeidler-Erdely (paz9@cdc.gov)*

*Pathology and Physiology Research Branch, †Laboratory for Occupational Cardiovascular Toxicology, [‡]Toxicology and Molecular Biology Branch and [§]Biostatistics and Epidemiology Branch, Health Effects Laboratory Division, National Institute for Occupational Safety and Health, Morgantown, WV

[¶]Deceased

"The findings and conclusions in this report are those of the author(s) and do not necessarily represent the views of the National Institute for Occupational Safety and Health"

Corresponding author: Aaron Erdely, PhD
e-mail: efi4@cdc.gov

Supplemental Material, Table S1: Genes comprising highlighted networks in whole blood cells

Whole Blood Cells	***Fold Change***	***p value***
4hr – "organismal development, infection mechanism, antimicrobial response"		
AIMP1	1.134	0.01837
ARRDC4	-1.106	0.04899
COMMD1	1.107	0.04396
EOMES	1.103	0.00983
F5	-1.248	0.01055
IFI27	1.364	0.00318
IFIT3	1.229	0.03232
IFITM3	1.478	0.00191
IKBKAP	-1.118	0.02758
IRF7	2.241	0.000001
ISG15	1.956	0.00108
OAS1	1.116	0.03961
OASL	1.141	0.00626
SLPI	-1.289	0.03268
SP100	1.206	0.00658
Tgtp1	1.175	0.00057
TLR7	1.146	0.04911
TMEM154	-1.101	0.01724
TNFAIP2	-1.126	0.04494
TRAFD1	1.152	0.04786
Trim30a/Trim30d	1.155	0.00919
VWF	-1.319	0.01835
28d – "organismal development, infection mechanism, antimicrobial response"		
AES	-1.274	0.03063
CAMK2B	-1.121	0.00020
DHX58	1.120	0.02289
DNTTIP1	1.179	0.00154
FLII	-1.143	0.03756
HLA-B	1.193	0.03445
IFI27	1.331	0.00595
IFI27L2	1.914	0.00505
IFI35	1.161	0.01134
IRF7	1.862	0.00003
ISG15	1.817	0.00300
ISG20	-1.335	0.04219
LY96	1.175	0.01304
NUB1	-1.161	0.03025
OAS1	1.254	0.00014
OAS2	1.232	0.01725
RAB31	-1.189	0.04853
SELPLG	-1.328	0.04275
SUMO1	1.125	0.01659
TLR7	1.158	0.03480
TRAF7	1.174	0.00619
Trim30a/Trim30d	1.159	0.00799
UBE2L3	-1.109	0.03288
USP18	1.344	0.00788
ZFHX2	-1.104	0.00594

Supplemental Material, Table S2: The most differentially expressed genes by fold change in the blood and aorta at 4 hr and 28 d post-inhalation exposure.

	**4hr – Fold Change**				**28d – Fold Change**			
	Increase		***Decrease***		***Increase***		***Decrease***	
Blood	*Irf7*	2.241	*Mmp9*	1.502	*Ifi27l2a*	1.914	*Ctsw*	1.372
	Oasl2	2.021	*Slc40a1*	1.423	*Irf7*	1.862	*Tubb1*	1.371
	Isg15	1.956	*Tgfbi*	1.355	*Oasl2*	1.856	*Plagl2*	1.370
	Ifitm3	1.478	*Vwf*	1.319	*Isg15*	1.817	*Aip*	1.355
	Lgals3bp	1.473	*Cdk13*	1.309	*Usp18*	1.344	*Isg20*	1.335
	Ppp1r15a	1.460	*Slpi*	1.289	*Ifi27l1*	1.331	*Selplg*	1.328
	Rnf114	1.415	*F5*	1.248	*Atox1*	1.316	*Wbp11*	1.327
	Usp18	1.385	*Myh9*	1.241	*Shfm1*	1.304	*Thy1*	1.302
	Ifi27	1.364	*Isy1*	1.210	*Clcn3*	1.270	*Ssc24b*	1.296
	H2-d1	1.331	*Lbr*	1.191	*Oas1b*	1.254	*Klf7*	1.295
Aorta	*Lgals3bp*	2.091	*Tfrc*	1.273	*Lgals3bp*	1.586	*Pln*	1.468
	Gbp4	1.904	*Ivns1abp*	1.199	*Thsd4*	1.555	*Hist4h4*	1.386
	Irgm2	1.660	*Ndufaf4*	1.196	*Klf6*	1.359	*Car3*	1.298
	Ifi27l2a	1.586	*Slc6a6*	1.165	*Mylk*	1.333	*Nnmt*	1.239
	Ifit3	1.555	*Atp5c1*	1.151	*Myh11*	1.312	*Adipoq*	1.193
	Usp18	1.533	*Large*	1.145	*Actn1*	1.247	*Acot13*	1.188
	Rsad2	1.411	*Acss1*	1.143	*Csrp1*	1.240	*Ndufs8*	1.187
	Isg15	1.360	*Cct4*	1.141	*Nox4*	1.230	*Mrps15*	1.187
	H2-Aa	1.335	*Cops2*	1.130	*Fblim1*	1.229	*Ffar2*	1.187
	Irf9	1.306	*Btbd1*	1.125	*Golim4*	1.227	*Cidec*	1.181

Supplemental Material, Table S3: Genes comprising highlighted networks in aorta

Aorta	***Fold Change***	***p value***
4hr – "antimicrobial response, inflammatory response, post-translational modification"		
EIF2AK2	1.188	0.00705
GBP4	1.904	0.00837
HLA-B	1.179	0.01133
HLA-C	1.148	0.00368
HLA-DQA1	1.335	0.03372
HLA-E	1.256	0.00735
IFI27L2	1.586	0.00006
IFIT2	1.102	0.03688
IFIT3	1.555	0.01550
IRF7	1.109	0.01657
IRF9	1.306	0.00870
ISG15	1.360	0.01873
LGALS3BP	2.091	0.00139
PIM3	1.157	0.00834
RPS2	-1.120	0.00525
RSAD2	1.411	0.04881
Trim30a/Trim30d	1.110	0.01709
USP18	1.533	0.01701

Supplemental Material, Table S4: Genes comprising highlighted networks in lung

Lung	*Fold Change*	*p value*
4hr – "dermatological diseases and conditions, genetic disorders, antimicrobial response"		
ADAR	1.452	2.17E-09
CASP1	1.317	8.13E-04
CYBA	1.371	2.24E-05
ELN	1.325	5.50E-03
FAM198B	-1.369	1.39E-07
GBP2	1.660	1.49E-04
GDF10	-1.446	1.17E-06
HSD11B1	-1.404	1.01E-06
IFI27L2	5.136	7.21E-12
IFI30	1.310	2.45E-05
IFI35	1.453	2.50E-06
Ifi47	2.290	4.48E-05
IFIH1	1.306	1.16E-07
IFIT2	3.257	5.01E-09
IFIT3	4.685	3.05E-10
IFITM3	1.640	2.69E-06
IRF7	4.730	2.57E-10
IRF9	1.833	2.11E-08
ISG15	7.401	5.15E-11
LGALS3	1.680	1.50E-11
LGALS3BP	4.675	1.56E-13
LRAT	-1.731	6.83E-05
MX1	2.076	2.69E-09
NOXO1	1.438	1.37E-05
OAS2	3.340	2.07E-09
Oasl2	4.552	7.04E-12
PPIC	1.408	9.60E-07
SERPINF1	-1.327	4.22E-07
TAP1	1.604	2.05E-05
**28d – "*antimicrobial response, inflammatory response, infection mechanism*"**		
CD83	1.743	1.55E-09
CTLA4	1.505	4.96E-08
DDX58	1.630	2.42E-07
DHX58	2.157	2.35E-08
GDF15	1.523	4.73E-14
GPC3	-1.343	3.20E-07
H2-Q8	1.767	3.67E-03
HLA-DQB1 (includes others)	1.628	2.39E-04
HLA-DRB1	1.449	4.11E-04
IFI30	1.556	6.74E-09
IFI35	1.338	8.09E-05
IFIT3	4.678	3.12E-10
IRF7	3.647	1.02E-08
IRF9	1.710	1.91E-07
ISG15	4.593	1.33E-08
LAMP3	1.473	6.97E-07
Ly6a (includes others)	1.365	9.82E-08
MZB1	1.538	9.51E-03

OASL	1.881	3.79E-07
OSMR	1.396	3.56E-08
RSAD2	4.527	4.26E-08
STAT2	1.573	1.88E-06

Supplemental Material, Table S5: Serum analytes contained in RodentMAP v2.0. * p<0.05 vs respective AIR sham.

Analytes	Units	4hr (n=4/group)		28d (n=4/group)	
		AIR	GMA-SS	AIR	GMA-SS
Apo A1 (Apolipoprotein A1)	ug/mL	45.2±4.1	43.7±1.4	37.5±0.9	35.9±0.5
CD40	pg/mL	90.7±6.0	95.8±10.4	96.2±3.8	106.6±5.5
CD40 Ligand	pg/mL	436.0±26.6	500.0±48.6	1970.0±206.7	1605.0±40.9
CRP (C Reactive Protein)	ug/mL	6.5±0.3	5.7±0.1*	5.5±0.3	6.2±0.6
EGF (Epidermal Growth Factor)	pg/mL	33.0±6.4	42.0±4.1	62.5±7.7	44.7±9.8
Endothelin-1	pg/mL	37.0±8.3	58.1±5.8	50.1±4.0	40.6±6.2
Eotaxin	pg/mL	990.0±65.7	979.8±48.4	563.0±42.7	470.3±38.0
Factor VII	ng/mL	8.9±0.6	9.9±0.5	41.7±2.4	36.2±3.8
FGF-9 (Fibroblast Growth Factor-9)	ng/mL	BLD	BLD	BLD	BLD
FGF-basic (Fibroblast Growth Factor-basic)	ng/mL	4.8±0.1	4.2±0.5	12.2±0.3	10.9±0.4*
Fibrinogen	ug/mL	n/a	n/a	n/a	n/a
GCP-2 (Granulocyte Chemotactic Protein-2)	ng/mL	35.0±2.9	36.4±1.6	26.3±4.2	21.2±4.1
GM-CSF (Granulocyte Macrophage-Colony Stimulating Factor)	pg/mL	BLD	BLD	BLD	BLD
GST-alpha (Glutathione S-Transferase alpha)	ng/mL	BLD	BLD	BLD	BLD
Haptoglobin	ug/mL	42.0±9.3	28.6±3.5	24.5±2.0	28.1±7.8
IFN-gamma (Interferon-gamma)	pg/mL	BLD	BLD	7.5±1.6	7.0±1.5
IgA (Immunoglobulin A)	ug/mL	19.7±2.5	35.8±9.7	34.0±1.9	26.5±1.8*
IL-10 (Interleukin-10)	pg/mL	352.3±6.3	324.5±24.8	BLD	BLD
IL-11 (Interleukin-11)	pg/mL	BLD	BLD	BLD	BLD
IL-12p70 (Interleukin-12p70)	ng/mL	BLD	BLD	BLD	BLD
IL-17 (Interleukin-17)	ng/mL	BLD	BLD	BLD	BLD
IL-18 (Interleukin-18)	ng/mL	4.0±0.1	4.3±0.2	14.4±1.1	16.1±1.4
IL-1alpha (Interleukin-1alpha)	pg/mL	227.0±17.4	209.3±34.4	212.3±74.4	198.0±35.8
IL-1beta (Interleukin-1beta)	ng/mL	8.9±0.4	8.8±0.4	17.2±0.6	17.2±0.3
IL-2 (Interleukin-2)	pg/mL	BLD	BLD	BLD	BLD
IL-3 (Interleukin-3)	pg/mL	BLD	BLD	BLD	BLD
IL-4 (Interleukin-4)	pg/mL	BLD	BLD	BLD	BLD
IL-5 (Interleukin-5)	ng/mL	1.2±0.1	1.2±0.1	0.2±0.0	0.3±0.0

IL-6 (Interleukin-6)	pg/mL	BLD	BLD	BLD	BLD
IL-7 (Interleukin-7)	ng/mL	BLD	BLD	BLD	BLD
IP-10 (Inducible Protein-10)	pg/mL	48.6±7.1	69.5±11.8	62.4±3.6	92.7±16.9
KC/GROalpha (Melanoma Growth Stimulatory Activity Protein)	ng/mL	BLD	BLD	BLD	BLD
LIF (Leukemia Inhibitory Factor)	pg/mL	707.3±12.1	632.5±16.2*	1545.0±76.9	1610.0±91.7
Lymphotactin	pg/mL	97.9±6.3	99.0±10.7	91.3±5.5	87.9±6.0
MCP-1 (Monocyte Chemoattractant Protein-1)	pg/mL	58.1±4.0	71.6±6.4	70.3±9.9	65.3±12.0
MCP-3 (Monocyte Chemoattractant Protein-3)	pg/mL	138.3±12.4	146.3±15.8	105.5±12.2	115.9±19.4
MCP-5 (Monocyte Chemoattractant Protein-5)	pg/mL	21.7±2.7	21.7±2.1	18.3±1.1	20.2±1.8
M-CSF (Macrophage-Colony Stimulating Factor)	ng/mL	6.3±0.2	6.1±0.2	7.0±0.2	7.0±0.1
MDC (Macrophage-Derived Chemokine)	pg/mL	806.8±22.8	786.8±32.8	627.3±50.1	563.8±51.5
MIP-1alpha (Macrophage Inflammatory Protein-1alpha)	ng/mL	0.8±0.1	0.8±0.0	1.8±0.1	1.9±0.0
MIP-1beta (Macrophage Inflammatory Protein-1beta)	pg/mL	42.3±6.0	58.0±13.4	99.3±2.2	96.8±6.8
MIP-1gamma (Macrophage Inflammatory Protein-1gamma)	ng/mL	21.0±1.1	19.2±1.3	16.3±0.4	17.4±0.6
MIP-2 (Macrophage Inflammatory Protein-2)	pg/mL	18.9±0.3	20.3±1.5	19.3±2.7	15.4±1.7
MIP-3beta (Macrophage Inflammatory Protein-3beta)	ng/mL	1.5±0.0	1.5±0.0	3.1±0.2	3.0±0.1
MMP-9 (Matrix Metalloproteinase-9)	ng/mL	152.8±12.5	112.7±9.2*	128.6±12.7	173.3±16.4
MPO (Myeloperoxidase)	ng/mL	57.1±3.3	48.8±1.3	59.3±3.2	62.6±1.4
Myoglobin	ng/mL	8.0±0.1	7.3±0.6	9.5±0.7	8.5±0.5
OSM (Oncostatin M)	ng/mL	0.08±0.00	0.10±0.00*	BLD	BLD
RANTES (Regulation Upon Activation, Normal T-Cell Expressed and Secreted)	pg/mL	BLD	BLD	BLD	BLD
SAP (Serum Amyloid P)	ug/mL	23.7±2.2	23.1±1.1	21.1±1.1	19.5±1.4
SCF (Stem Cell Factor)	pg/mL	257.0±17.1	246.8±14.2	253.8±15.0	235±1.9
SGOT (Serum Glutamic-Oxaloacetic Transaminase)	ug/mL	15.1±0.8	18.0±4.2	70.1±5.1	70.9±5.9
TIMP-1 (Tissue Inhibitor of Metalloproteinase Type-1)	ng/mL	1.1±0.3	1.1±0.1	0.7±0.0	1.0±0.2
Tissue Factor	ng/mL	5.3±0.3	5.2±0.3	13.0±0.7	12.0±0.4
TNF-alpha (Tumor Necrosis Factor-alpha)	ng/mL	BLD	BLD	BLD	BLD
TPO (Thrombopoietin)	ng/mL	52.7±1.9	51.4±3.6	106.0±1.1	109.3±1.7
VCAM-1 (Vascular Cell Adhesion Molecule-1)	ng/mL	1635.0±52.7	1590.0±101.6	1715.0±18.5	1890.0±23.8*
VEGF (Vascular Endothelial Cell Growth Factor)	pg/mL	179.0±11.3	178.5±14.8	153.5±9.4	140.0±3.1
vWF (von Willebrand Factor)	ng/mL	127.8±5.0	123.5±1.7	91.5±4.4	88.9±1.6

Supplemental Material, Table S6: Genes associated with PRR canonical pathway in lung (Figure S7).

	**4hr**		**28d**	
Gene	***Fold Change***	***p value***	***Fold Change***	***p value***
C3	1.504	1.74E-04	1.692	6.10E-06
C1qa	1.993	4.72E-09	2.189	3.59E-10
C1qb	2.571	4.24E-10	2.966	2.11E-11
C1qc	2.383	1.69E-09	2.757	6.66E-11
Casp1	1.317	8.13E-04	1.599	7.24E-07
Ccl5	1.807	4.14E-04	1.536	6.94E-03
Clec7a	1.440	1.49E-04	1.810	1.10E-07
Ddx58	1.773	1.38E-08	1.630	2.42E-07
Eif2ak2	2.493	3.71E-09	2.206	5.70E-08
Ifih1	1.306	1.16E-07		
Il1b			2.007	4.27E-05
Irf7	4.730	2.57E-10	3.647	1.02E-08
Myd88	1.348	1.66E-05	1.364	9.59E-06
(Oas2)	3.340	2.07E-09	3.301	2.52E-09
Tlr2	1.658	1.97E-07	1.791	1.48E-08
Tlr7			1.369	1.05E-05

Supplemental Material, Table S7: Bronchoalveolar lavage parameters in C57BL/6J mice up to 28 d following 10 d of GMA-SS welding fume inhalation. * $p<0.05$ vs sham.

		1d	***14d***	***28d***
LDH (U/L)	AIR	47±4	66±2	52±3
	GMA-SS	213±13*	214±13*	209±11*
Albumin (mg/ml)	AIR	0.10±0.01	0.12±0.01	0.13±0.01
	GMA-SS	0.36±0.03*	0.32±0.03*	0.35±0.02*
PMN (10^4)	AIR	0.00±0.00	0.10±0.10	0.77±0.50
	GMA-SS	6.43±1.95*	44.38±6.58*	41.66±4.49*
Macrophages (10^5)	AIR	3.83±0.53	3.07±0.28	3.30±0.40
	GMA-SS	15.75±1.63*	19.70±1.32*	31.77±2.70*
Lymphocytes (10^4)	AIR	0.19±0.05	0.14±0.56	0.28±0.11
	GMA-SS	11.95±2.88*	6.68±2.48	11.66±2.85*

Mice were exposed to GMA-SS by inhalation for 10 d at 40 mg/m^3 for 3 hr/day and harvested at days 1, 14 and 28 d post-exposure. For bronchoalveolar lavage (BAL) collection, 0.6 mL of cold Ca^{+2} and Mg^{+2} free PBS was instilled into the lung and then withdrawn in anesthetized mice (first fraction BAL). Two subsequent lavages (1.0 mL each) were then collected. The supernatant of the first fraction was used to determine lactate dehydrogenase (LDH) and albumin. The cell pellet of both fractions was combined, resuspended in PBS, and used for cell enumeration and differential staining [3]. PMN = polymorphonuclear cells

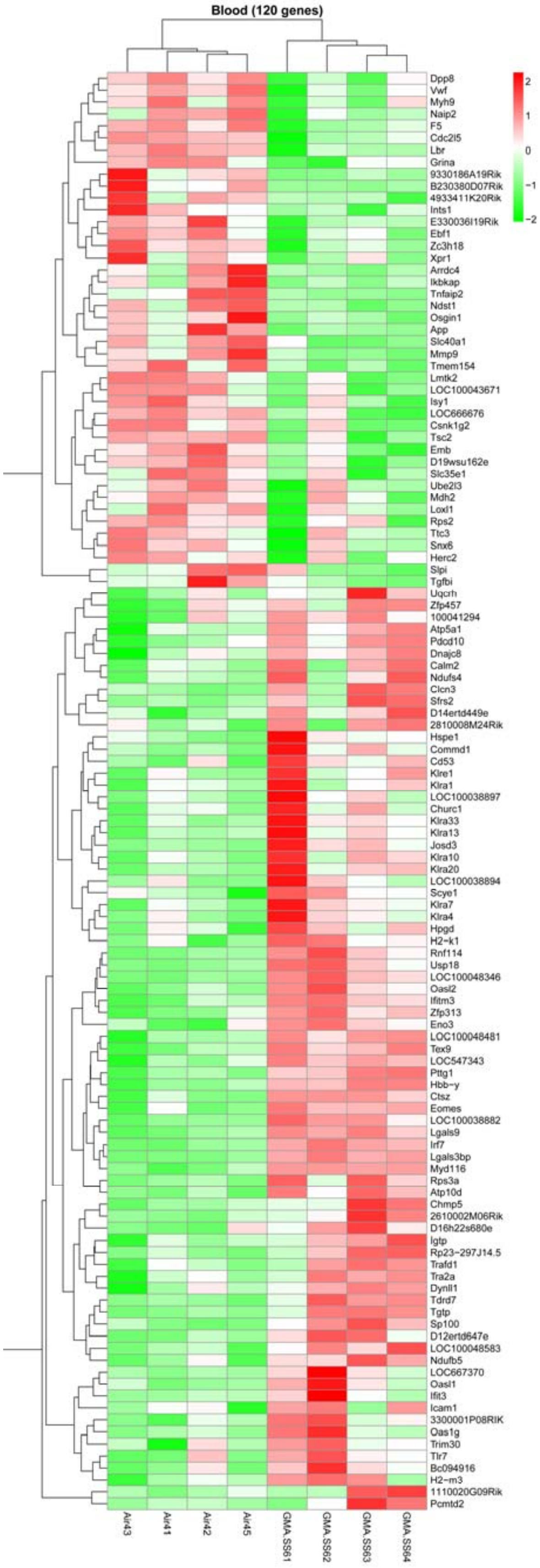

Supplemental Material, Figure S1: Hierarchial clustering analysis of differentially expressed genes in the blood of C57BL/6J mice exposed to GMA-SS welding fume or air at 4 hr post-exposure after p-value and fold change adjustment ($p<0.05$ and fold change of +/- 1.1). The range of gene expression values are represented as the color scheme green-white-red which indicates low-moderate-high gene expression compared to the corresponding air control.

Supplemental Material, Figure S2: The network "organismal development, infection mechanism, antimicrobial response" in blood at 4hr. The network contained 22 of 35 focus molecules with a score of 44.

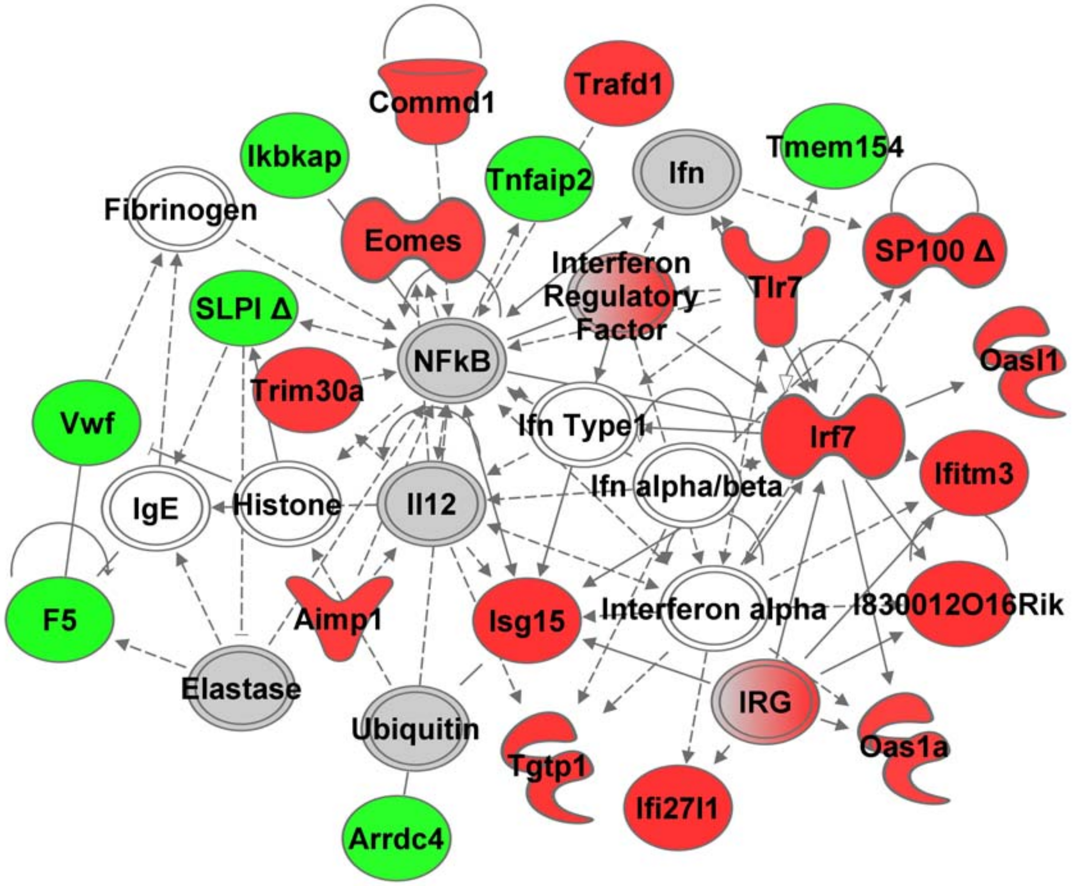

© 2000-2011 Ingenuity Systems, Inc. All rights reserved.

Supplemental Material, Figure S3: Functional analysis for whole blood cells at 28d.

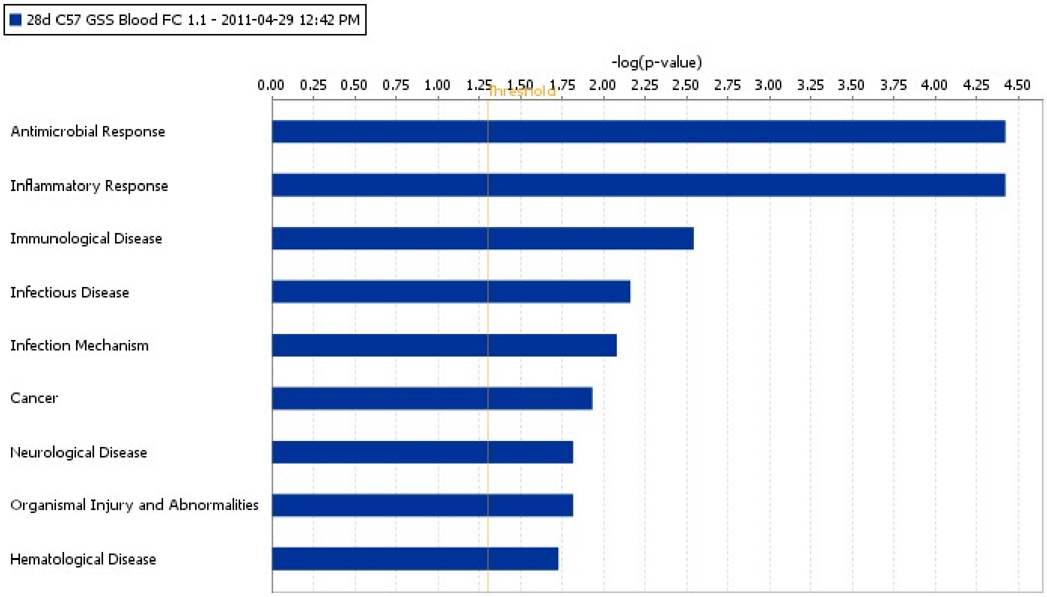

© 2000-2011 Ingenuity Systems, Inc. All rights reserved.

Supplemental Material, Figure S4: Functional analysis for aorta at 4hr

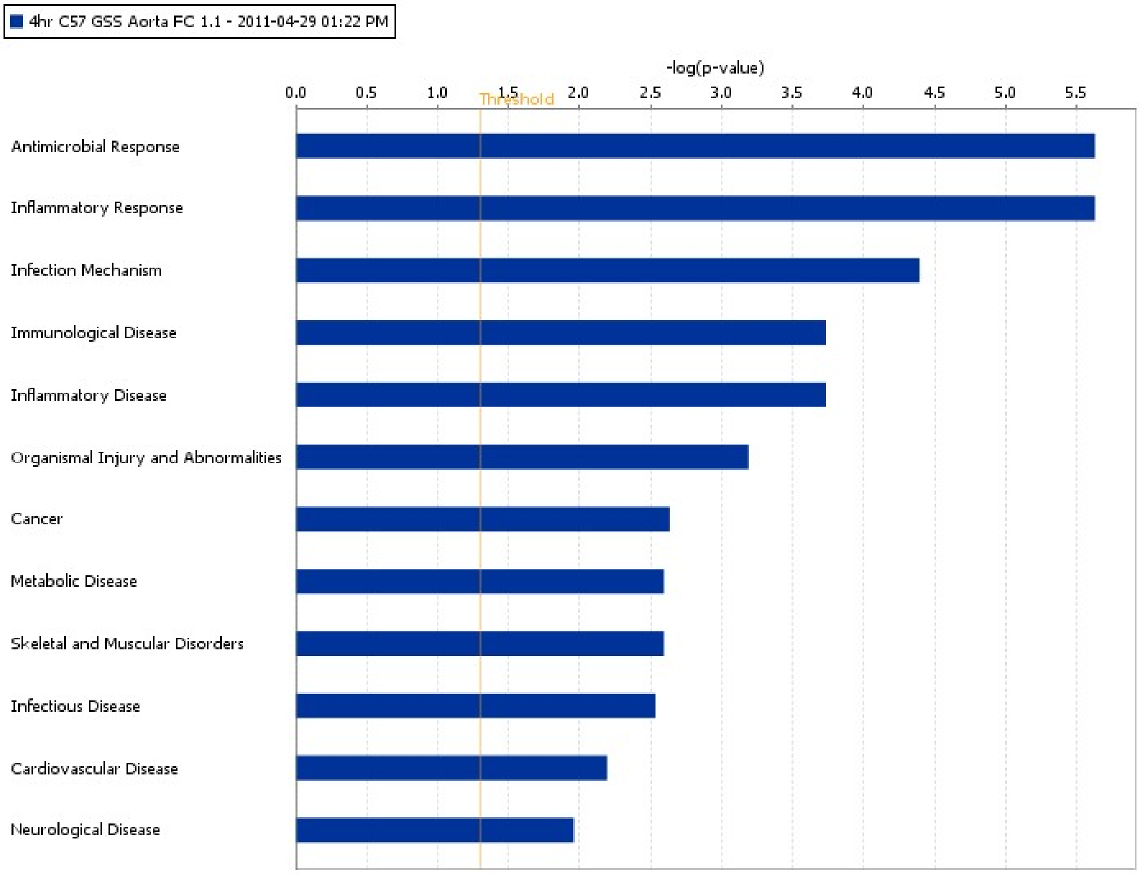

© 2000-2011 Ingenuity Systems, Inc. All rights reserved.

Supplemental Material, Figure S5: The network "dermatological diseases, genetic disorder, antimicrobial response" in lung at 4hr. The network contained 29 of 35 focus molecules with a score of 45.

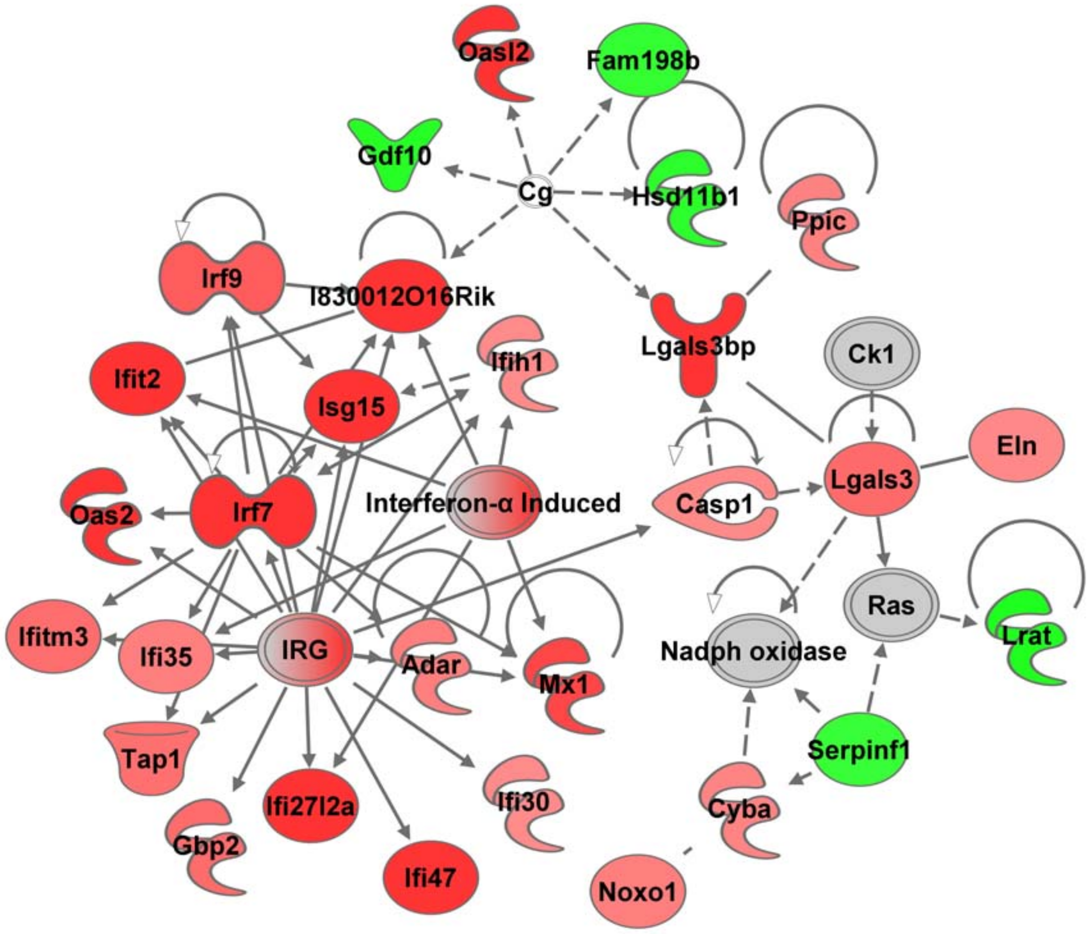

© 2000-2011 Ingenuity Systems, Inc. All rights reserved.

Supplemental Material, Figure S6: Canonical pathway “activation of IRF by cytosolic PRR” in lung at 28d (p=8.6E-06).

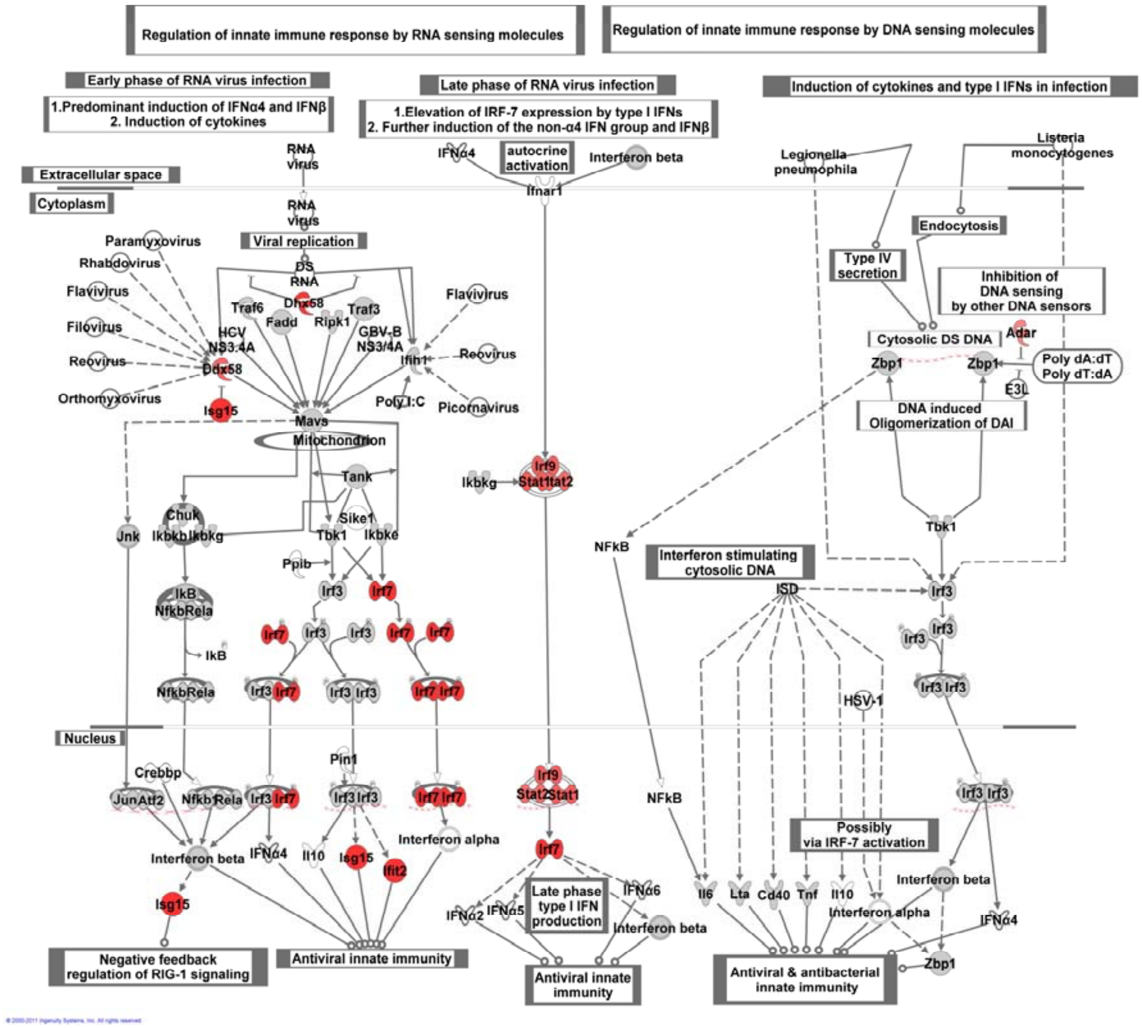

Supplemental Material, Figure S7: Canonical pathway "role of PRR in recognition of bacteria and viruses" in lung at 28d (p=1.11E-09).

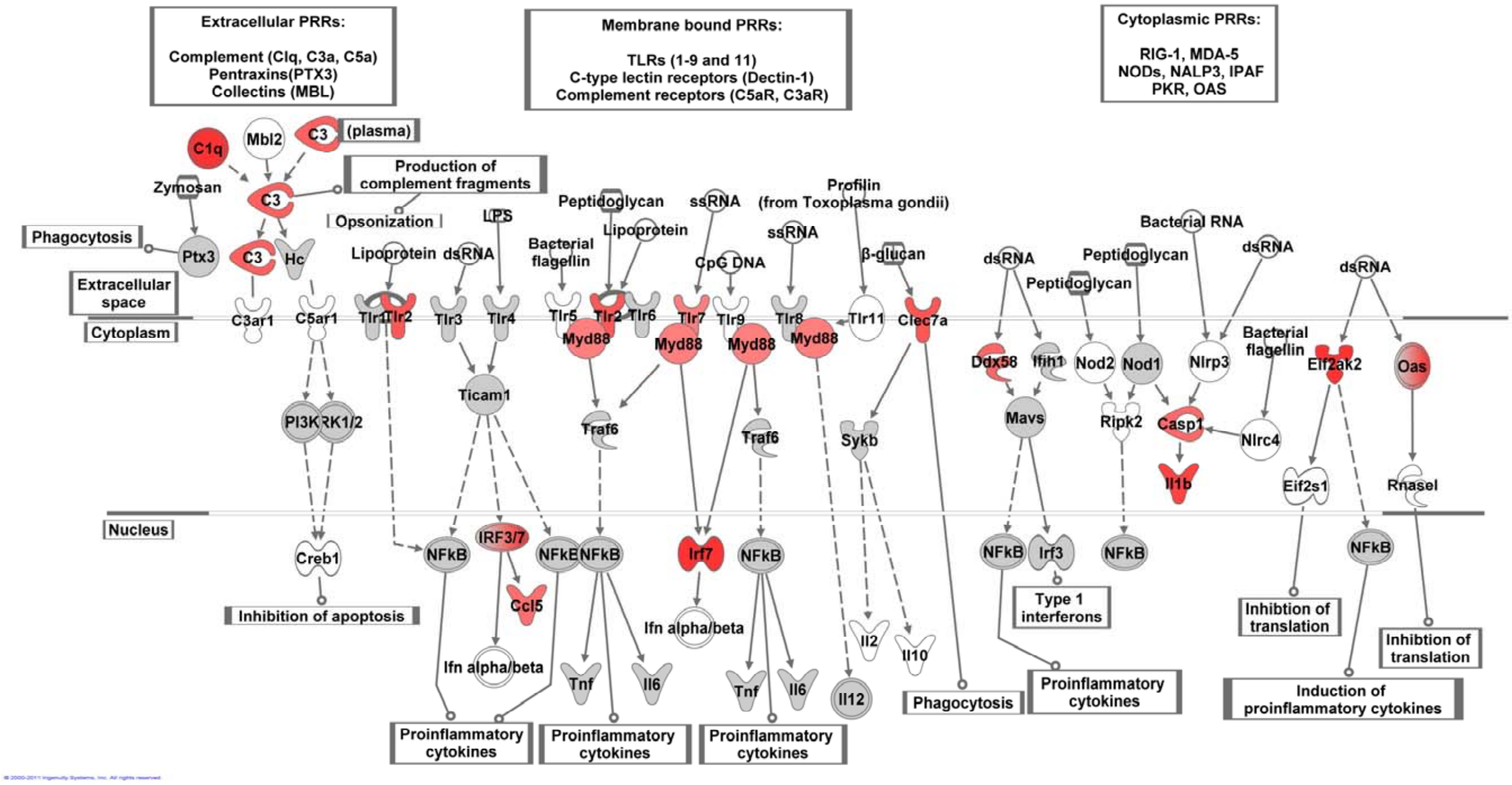

Supplemental Material, Figure S8: *Nlrp3* and associated gene expression by qRT-PCR in lung at 4 hr and 28 d. * $p<0.05$ vs sham.

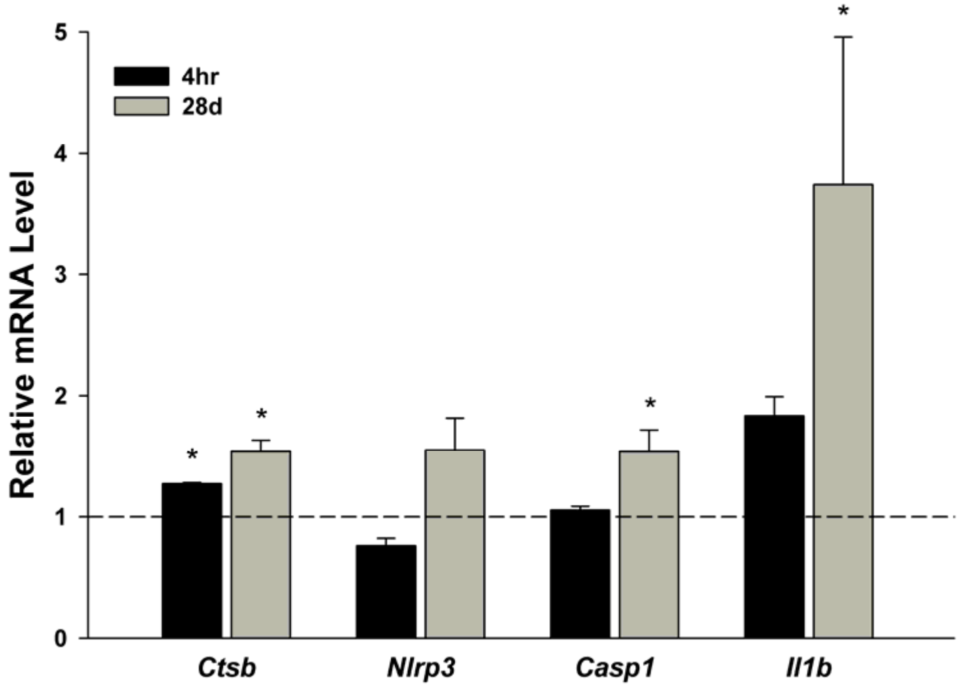

Supplemental Material, Figure S9: Cytotoxicity measured in bronchoalveolar lavage (BAL) and relative mRNA expression of inflammatory markers in the lung after various particle exposures. Male C57BL/6J mice were exposed to 340 µg of gas metal arc – stainless steel (GMA-SS), 340 µg gas metal arc – mild steel (GMA-MS), 40 µg of multi-walled carbon nanotubes (MWCNT), or 40 µg single-walled carbon nanotubes (SWCNT) (n=5 or 6 per group for all exposures) by pharyngeal aspiration and BAL was collected 24 hr post-exposure. Cytotoxicity **(A)** was measured by lactate dehydrogenase (LDH) activity and represented as percent (%) change from control (* $p<0.05$). Specific inflammatory genes **(B)** represented include metallothionein 2 (*Mt2*), chemokine (C-X-C) motif ligand 1 (*Cxcl1*), interleukin-6 (*Il6*) and colony stimulating factor 3 (granulocyte) (*Csf3*). All values were significantly increased from respective sham.

A:

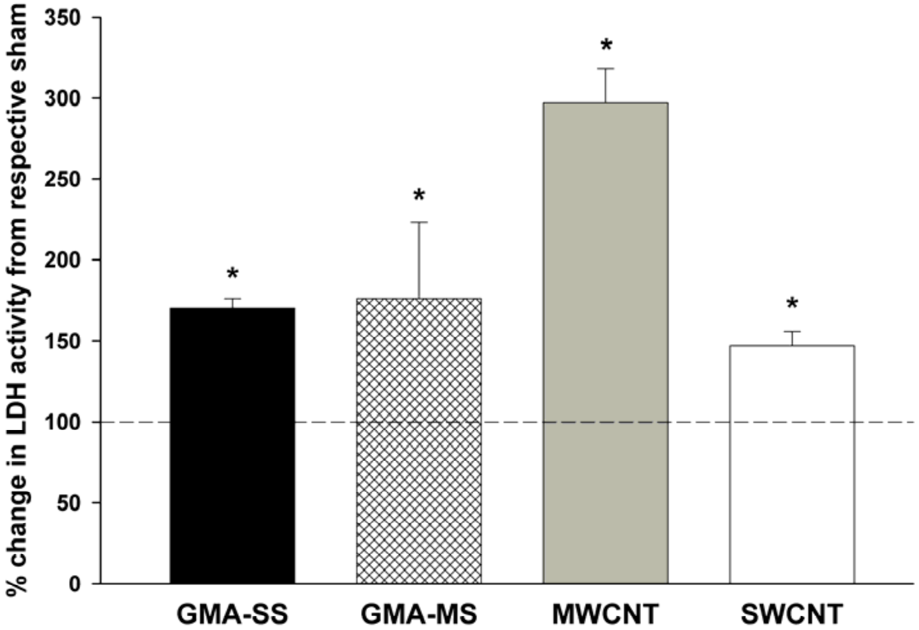

B:

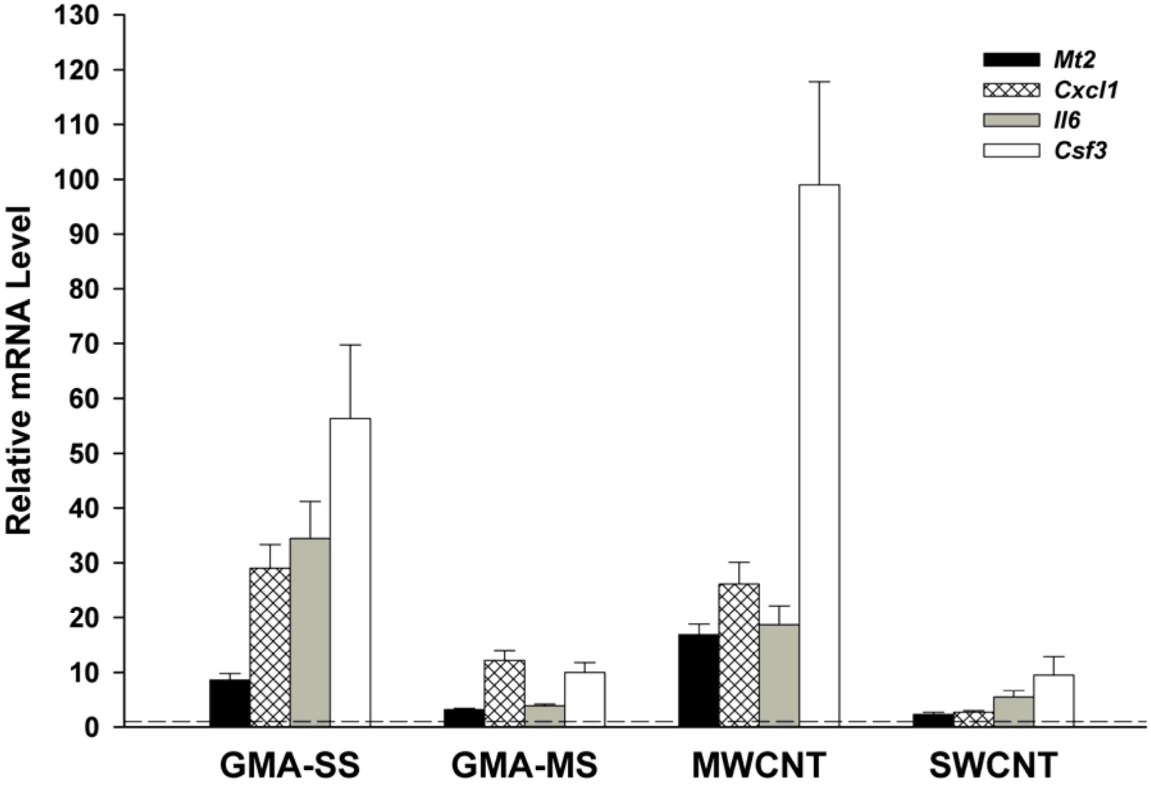
Relative mRNA Level
130
120
110
100
90
80
70
60
50
40
30
20
10
0
Mt2
Cxcl1
Il6
Csf3
GMA-SS
GMA-MS
MWCNT
SWCNT

Supplemental Material, Figure S10: Legend to network pathway shapes

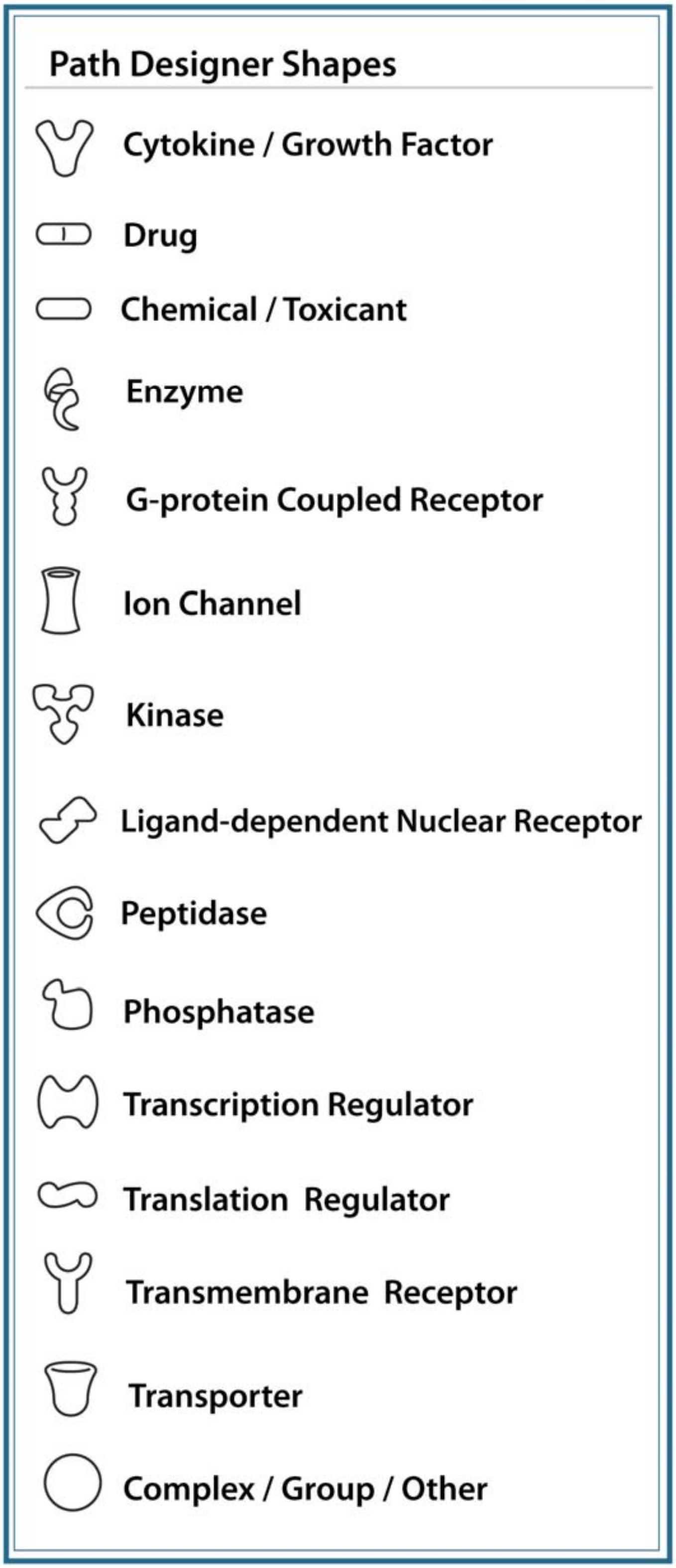

Supplement: Additional file 1 — Contains additional data to supplement the microarray and pathway analysis data presented in the results section. [file 1743-8977-9-25-S1.pdf]
